# Dietary Nutrients, Proteomes, and Adhesion of Probiotic Lactobacilli to Mucin and Host Epithelial Cells

**DOI:** 10.3390/microorganisms6030090

**Published:** 2018-08-21

**Authors:** Hasan Ufuk Celebioglu, Birte Svensson

**Affiliations:** 1 Department of Biotechnology, Bartin University, 74110 Bartin, Turkey; ufukcelebioglu@gmail.com; 2 Department of Biotechnology and Biomedicine, Technical University of Denmark, DK-2800 Kgs Lyngby, Denmark

**Keywords:** lactobacilli, probiotics, adhesion, mucin, intestinal cells, carbon sources, polyphenols, surface proteomes, protein identification, moonlighting proteins

## Abstract

The key role of diet and environment in human health receives increasing attention. Thus functional foods, probiotics, prebiotics, and synbiotics with beneficial effects on health and ability to prevent diseases are in focus. The efficacy of probiotic bacteria has been connected with their adherence to the host epithelium and residence in the gut. Several in vitro techniques are available for analyzing bacterial interactions with mucin and intestinal cells, simulating adhesion to the host in vivo. Proteomics has monitored and identified proteins of probiotic bacteria showing differential abundance elicited in vitro by exposure to food components, including potential prebiotics (e.g., certain carbohydrates, and plant polyphenols). While adhesion of probiotic bacteria influenced by various environmental factors relevant to the gastrointestinal tract has been measured previously, this was rarely correlated with changes in the bacterial proteome induced by dietary nutrients. The present mini-review deals with effects of selected emerging prebiotics, food components and ingredients on the adhesion of probiotic lactobacilli to mucin and gut epithelial cells and concomitant abundancy changes of specific bacterial proteins. Applying this in vitro synbiotics-like approach enabled identification of moonlighting and other surface-located proteins of *Lactobacillus acidophilus* NCFM that are possibly associated with the adhesive mechanism.

## 1. Introduction

Diet and its effects on the microbiota of the gastrointestinal tract (GIT) are of paramount importance for human health through influence on the immune system, and hence allergies, diabetes, bowel disorders, and cancers. Such an increasingly recognized role of food components may eventually invoke suggestions to counteract the adverse effects of inappropriate dietary regimes and risks to develop various illnesses. Awareness has thus improved, especially in the past decade, on consumption of functional foods and beverages with possible favorable impact on health via insights into the gut microbiota and microbiome [1,2,3,4]. This includes attention on conventional or everyday foods as part of a normal diet that contains natural compounds proven in scientific and clinical studies to exert positive effects on health beyond a mere nutritional value. The present mini-review focuses on molecular insights at the bacterial proteome level characterizing interactions of lactobacilli, dietary components, mucin, and host epithelial cells with specific emphasis on factors important in GIT adhesion [5,6,7,8] that may eventually be managed by food choices.

## 2. Gastrointestinal Tract, Mucins, and Lactobacilli

### 2.1. Mucosa and Mucins

**Figure 1:**
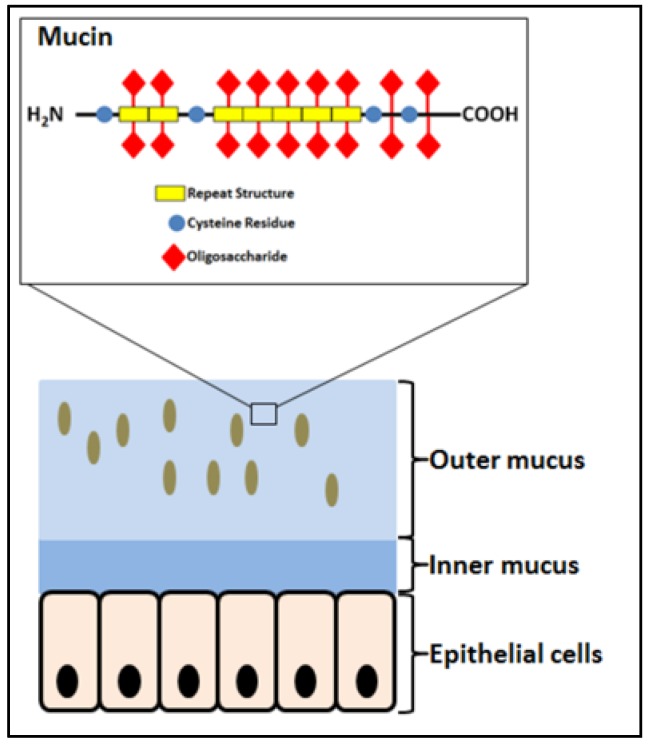
Schematics of the mucosa covering the gastrointestinal tract and the structure of mucin, the glycoprotein component of the mucus layer. Brown ovals illustrate bacteria. Modified from Reference [17].

The GIT is home to microorganisms and a major location for innate and acquired immunity [9,10,11,12,13]. It is covered by mucus that accommodates the majority of the microbiota and is the place for the attachment of bacteria, being either probiotic, commensal or pathogenic, and intimate encounters with the host. The mucus (Figure 1) is a gel layer serving for hydration and lubrication as well as a barrier against pathogens and harmful substances [14,15,16]. It consists mostly of water, glycoproteins, salts, and lipids. Mucins are large extracellular proteins, which are heavily glycosylated (about 80% of the mass) carrying numerous oligosaccharide decorations of DP 4–15 and providing viscosity and gelling properties [14,17,18]. The protein moieties of mucins possess (i) a central glycosylated region composed of sequence repeats rich in serine, threonine, and proline, (ii) N- and C-terminal regions of globular-like structure with relatively low glycosylation, and iii) cysteine-rich domains engaged in disulfide mediated dimerization and polymerization (Figure 1) [14,17,19].

### 2.2. Probiotic Lactobacilli

**Figure 2:**
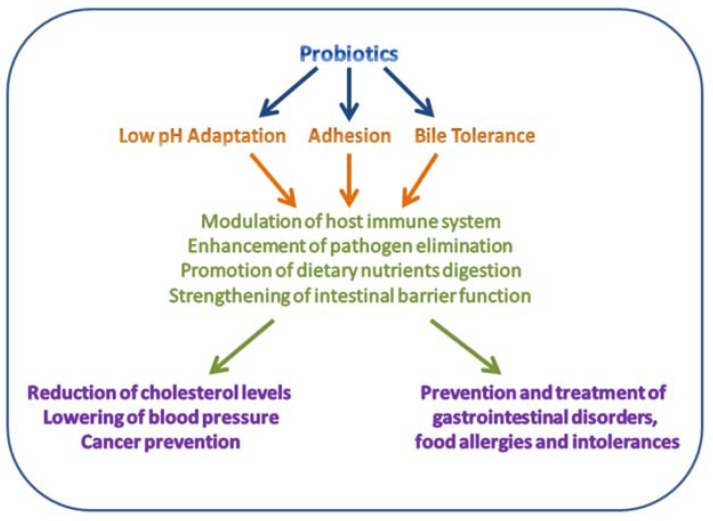
Some beneficial effects of probiotics to human health. Modified from Reference [29].

Lactobacilli are non-spore forming, Gram-positive, non-motile, rod-shaped bacteria varying in length and important members of a normal human microbiota [4,20]. Generally, lactobacilli are facultative anaerobes and able to grow in anaerobic and aerobic environments, producing lactic acid as end product of glucose fermentation [21]. The human GIT is colonized by several *Lactobacillus* species, including *Lactobacillus acidophilus*, *L. brevis*, *L. casei*, *L. fermentum*, *L. gasseri*, *L. johnsonii*, *L. paracasei*, *L. plantarum*, *L. rhamnosus*, and *L. salivarius* [22,23]. Some lactobacilli have been approved as probiotics defined by the FAO/WHO as “live microorganisms that, when administered in adequate amounts, confer a health benefit on the host” [24]. This distinguishes the live microorganisms directly applied for gaining favorable health effects from those used in processing of foods or production of various compounds [22,25,26]. The probiotic status requires that the bacterial species or strain is associated with two general host health benefits, namely support of (i) a healthy digestive tract and (ii) a healthy immune system (Figure 2) [24]. Other criteria for a probiotic bacterium include being safe (i.e., no gene transfer to or from other species or production of toxins), to survive through the GIT and adhere effectively to the mucosa, be viable during storage and use, be isolated from a human source, and have good sensory abilities [4,26,27,28].

Lactobacilli can exert health benefits in several ways, for example by inhibiting growth of pathogenic bacteria as they produce lactic, propionic, and acetic acids lowering the pH that suppresses proliferation of pathogens in the GIT [20,30,31]. Additionally, they can competitively prevent attachment of pathogens to the epithelium [16,32,33,34,35]. Lactobacilli have also been connected with improvements of gut diseases or disease-associated symptoms. Several studies thus reported that some lactobacilli can be successful in treating irritable bowel syndrome [3,4,36,37]. Different lactobacilli, either alone or in combination with other probiotics (e.g., bifidobacteria) have improved antibiotic-associated diarrhea [4,38]. Also, profiling of the crosstalk between bacteria and intestinal cells at the molecular level is getting increasing attention [39].

## 3. Lactobacillus Subproteome Analyses

The characteristics of protein profiles of probiotic bacteria with relevance for adhesion have been explored by applying proteomic approaches involving in silico as well as experimental procedures for identification and quantification of surface-exposed and secreted proteins [40,41,42]. Notably, experimental protocols are established dedicated to analysis of the surface subproteome and the secretome (exoproteome), respectively, both of which are of particular interest when describing the bacteria-host molecular interactions in adhesion processes. Surface proteins have thus been isolated from *L. acidophilus* NCFM and other lactobacilli by lithium chloride or guanidine hydrochloride treatment and identified by liquid chromatography and mass spectrometry (LC-MS) or by 2-dimensional electrophoresis (2-DE) and mass spectrometry [10,40,42,43,44,45,46,47]. Several *L. acidophilus* proteins including the surface layer protein SlpA have been identified as potential adhesive molecules by proteome analysis using 2-DE and mass spectrometry [42,44,45] and by comparison of strains with high and low adhesive capacity to Caco-2 intestinal cells [43]. Proteins involved in adhesion have also been monitored by analyzing subproteomes using western blotting [48].

## 4. Adhesion Factors of Lactobacilli

### 4.1. Assaying Bacterial Adhesion In Vitro

**Figure 3:**
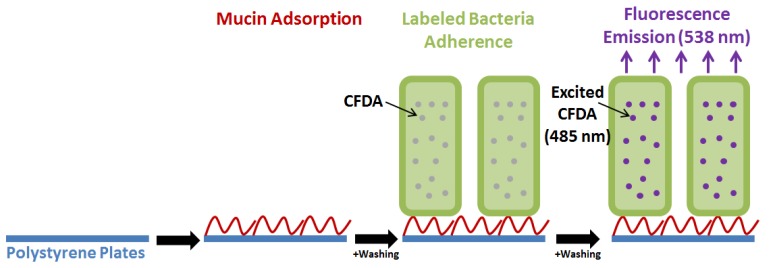
Mucin is adsorbed (or HT-29 cells are grown) onto polystyrene plates and added *Lactobacillus acidophilus* NCFM (OD at 600 nm = 0.5) labeled with carboxyfluorescein diacetate (CFDA). Following washing, the remaining fluorescence is determined after bacterial cell lysis as a measure of the amount of adhered bacteria [44,51].

Adherence of beneficial bacteria to the intestinal mucosa is considered important for exerting their function and is a claimed key characteristic of probiotics [5,15,28,49,50]. In vitro studies thus commonly analyze adherence of probiotic bacteria to i) mucin adsorbed onto abiotic surfaces (e.g., polystyrene), ii) confluent intestinal cell layer cultures (HT-29, HT-29-MTX and Caco-2 are customary cell lines used in adhesion assays) in cell/tissue culture plates, or iii) extracellular matrix components such as fibronectin and collagen [7,15,49,51,52,53] (Figure 3). Although these commonly applied cell lines are obtained from colonic carcinoma they express many of the markers associated with normal small intestine villus cells, including a diverse ability to produce or not produce mucin proteins [7,52,53].

Bacterial molecular factors that stimulate adhesion have been identified at the genome and proteome levels by comparing strains showing different adhesive capacity [43,54]. Subsequent validation can involve gene knock-out or overexpression, but methods relying on (i) depletion or shaving of proteins situated on the bacterial surface, (ii) effects in adhesion assays by competition with recombinantly produced proteins, or (iii) competition amongst bacteria for elimination of pathogens, also received broad interest [8,10,23,34,44,45,46,47,55].

Bacterial adhesion processes can be mediated by physico-chemical forces such as hydrophobic and electrostatic interactions via lipoteichoic acids or surface proteins [5,15,50]. Hydrophobicity is evaluated by mixing the bacterial culture with organic solvents and measuring the contents of bacteria in the aqueous phase spectrophotometrically at 600 nm before and after the solvent addition [56]. It has been suggested to apply hydrophobicity analysis as a screening tool in evaluating adhesion potential of probiotic bacteria [33,57]. Also bacterial aggregation has been connected with the adhesive ability [50] and claimed a desirable characteristic of probiotics, since aggregated bacteria inhibited adhesion of pathogenic bacteria [58]. Although this phenomenon is complex and the precise mechanism is unclear, aggregation-promoting proteins on the surface of bacteria were noted to be important. For example, deletion of an aggregation-promoting factor of *L. acidophilus* NCFM (LBA0493) reduced adhesion to Caco-2 cells, mucin, and the extracellular matrix components fibronectin, collagen IV, and laminin, even though the morphology of the bacterial cells was not altered [59].

### 4.2. Identification of Surface Proteins in Lactobacillus acidophilus NCFM

Not surprisingly, lactobacilli were reported to adhere to mucin and intestinal cells through their surface-associated proteins [7,11,47,59,60]. Thus, previously several surface proteins were identified to be involved in adhesion of lactobacilli and the findings were in some cases validated by using genetic tools, typically gene knock-out/suppression or overexpression [10,11,44,47,54,59,60]. Importantly, the growth state (e.g., logarithmic versus stationary phase) is connected with distinct surface-layer associated proteome differences [40]. This fact is significant as in vitro analyses for comparative effects on proteins in surface and extracellular proteomes with roles in adhesion should be done at a relevant growth stage. Also both environmental conditions (pH, bile) and carbon sources or additives (refer to Section 6), are important [61].

A prominent group of surface proteins in lactobacilli with a role in adhesion are mucus-binding proteins (MUBs) [15,17,19]. Their structures include repeated domains, called MucBP repeats, suggested to establish microbe-host interactions; MucBP containing proteins have been claimed to promote the evolution of lactobacilli to be primary gut microorganisms [15,17,62,63]. Other key factors are the surface layer protein (Slp), which is one of the dominant proteins in lactobacilli, and the surface-layer associated proteins (Slaps), forming a self-assembled paracrystalline monolayer that covers all of the bacterial surface [10,11,40,47]. Inactivation of the protein SlpA by gene knock-out in *L. acidophilus* NCFM decreased adhesion to Caco-2 cells by 84% compared to wild type [7]. Furthermore, insertional gene inactivation of fibronectin-binding protein A (LBA1148) and mucin-binding protein (LBA1392) decreased the adhesion of *L. acidophilus* NCFM by 76% and 65%, respectively [7]. Deletion of another *L. acidophilus* NCFM fibronectin-binding protein (FbpB, LBA0191) decreased adhesion to mucin and fibronectin by 47% and 72%, respectively [60]. The deletion of the serine protease homologue Prtx caused increased autoaggregation, and the Δ*prtx* strain showed 40% and 20% increased adhesion to mucin and fibronectin, respectively, compared to the parent [10]. Prtx likely impacts how proteins are displayed on the bacterial cell surface and may alter the structure and properties of the epithelial cell matrix. Notably, *L. acidophilus* NCFM Δ*prtx* improved the GIT epithelial barrier integrity in germ-free mice [10]. Overall, the general understanding is that multiple surface-associated bacterial proteins serve to adhere to mucin and intestinal cells.

**Figure 4:**
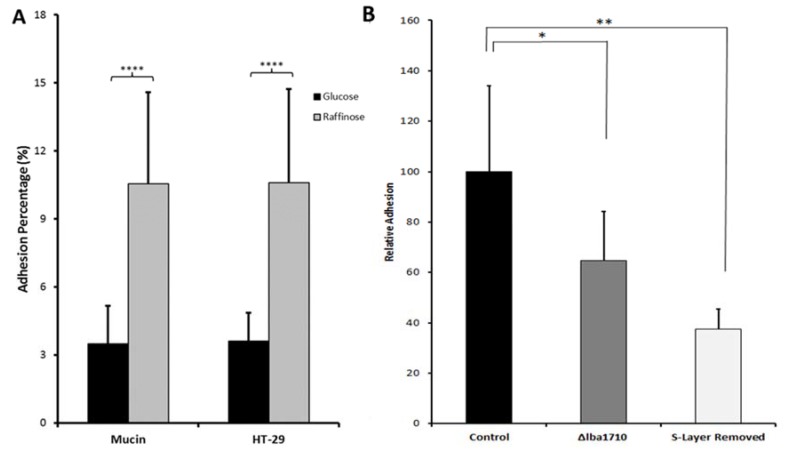
In vitro adhesion of *Lactobacillus acidophilus* NCFM to mucin and HT-29 cells. (**A**). Bacteria grown with glucose (black) and raffinose (grey). (**B**). Adsorption to mucin of wild type grown on raffinose (black), a thermostable pullulanase deletion mutant (Δlba1710) (grey), and surface depleted wild type (white). Modified from Reference [44].

Thermostable pullulanase from *Streptococcus pyogenes* binds to glycoproteins including submaxillary mucin and has a role in adhesion to epithelial cells, although its canonical function is to catalyze hydrolysis of α-1,6-glucosidic linkages in glycogen, amylopectin and pullulan [64,65]. Thus, a potential role in adhesion was confirmed for thermostable pullulanase from *L. acidophilus* NCFM by a pullulanase-deficient mutant (Δlba1710) causing 35% less adhesion to mucin than the wild type (Figure 4) [44]. In a different approach, the moonlighting *L. acidophilus* NCFM elongation factor G and pyruvate kinase, showing increased abundancy in the surface proteome when grown on raffinose, cellobiose, and glucose supplemented with either mucin or resveratrol (Table 1), were produced recombinantly and pre-incubated with a mucin layer, which reduced the bacterial adhesion by 8–13% [45]. Moonlighting proteins will be described in more detail below (refer to Section 4.4).

Furthermore, by using bioinformatics in silico analysis myosin cross-reactive antigen (LBA0649) and cell division protein A (LBA0223) have been assigned roles in adhesion of *L. acidophilus* NCFM to Caco-2 cells as demonstrated by corresponding insertional gene disruptions that caused 50 and 45% reduced adhesion for exponential and stationary phase cultures, respectively [8].

### 4.3. Some Adhesive Surface Proteins Identified in Other Probiotic Lactobacilli

In *L. rhamnosus* GG the SpaCBA pili protein was found to be important for adhesion and might confer prolonged GIT retention. This was supported by lack of adherence to Caco-2 cells of a non-piliated *L. rhamnosus* GR-1 strain as well as by a SpaCBA knock-out of *L. rhamnosus* GG [54]. In *L. rhamnosus* FSMM22 several anchorless surface proteins have been identified as laminin-adhesins, including known moonlighting proteins as well as ribosomal proteins [66] (refer to Section 4.4). Notably, on the host side, screening a Toll-like receptor-derived peptide library for improved adhesion of *L. rhamnosus* GG identified an octapeptide mediating enhanced adhesion to Caco-2 cells [67]. Indeed the interaction between bacteria and the host mucosa is gaining attention including analysis of effects at the molecular level reported by proteome analysis of co-cultures [39].

### 4.4. Identification of Moonlighting Proteins in Probiotic Lactobacilli

**Figure 5:**
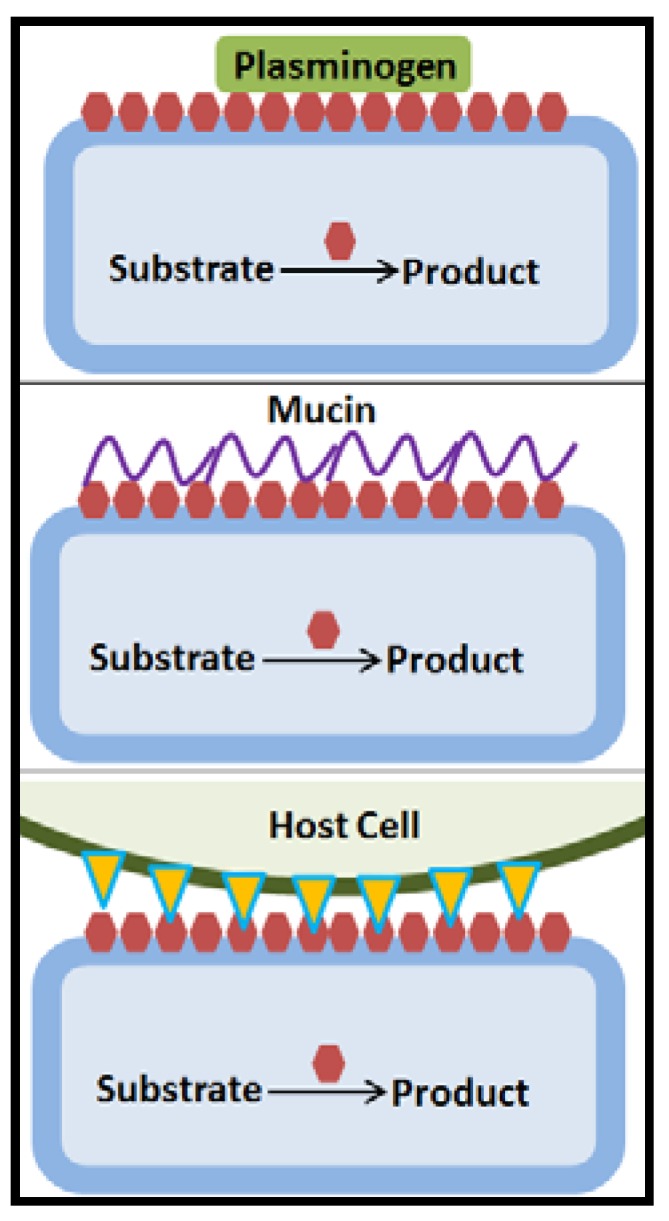
Moonlighting proteins (red hexagonals) have multiple functions, the main function for example being as housekeeping enzymes in the intracellular metabolism. The secretion mechanism to the cell surface where moonlighting proteins can act with roles in adhesion to proteins of the extracellular matrix or plasminogen, glycoproteins of the mucin layer or host cell surface proteins has not been elucidated [55,61,68,69,70].

Besides pili proteins, which are situated on the bacterial surface and have a canonical function in cell-cell and cell-extracellular matrix protein interactions, lactic acid bacteria contain a large number of proteins referred to as moonlighting proteins, which have been demonstrated to act in adhesion [16,66,68] (Figure 5). Moonlighting proteins are multifunctional proteins that participate in unrelated biological processes and which are not the result of gene fusion. They are anchorless and the mechanism of the export to the surface has not been identified, but it was proposed to occur eitherby non-canonical secretion, as these proteins lack a signal motif, or by binding of cytosolic proteins from lysed cells onto the cell wall of intact bacteria, although this latter mechanism recently received reduced support [5,61,68,69,70]. Moonlighting proteins comprise a subset of multifunctional proteins belonging to different categories, metabolic enzymes, molecular chaperones, translational elongation factors, ribosomal and other proteins [16,29,50,66,68,69,71]. Following simulated GIT passage by exposure to bile and acidic pH, *L. paracasei* CIDCA 83123 showed clearly increased capacity to adhere to mucin and Caco-2 cells. The proteome analysis indicated differentially abundant proteins, which increased by 1.4–4.3 fold, some of which were moonlighting proteins, glyceraldehyde-3-phosphate dehydrogenase (GADPH, two forms), L-lactate dehydrogenase, phosphoglyceromutase and UTP-glucose-1-phosphate uridyltransferase, while cell wall hydrolase, amylase family (two forms), surface antigen (two forms), a GADPH form, protein lacX, and galactose mutarotase-like protein decreased by 2–8 fold [61]. Notably, also GADPH of *L. acidophilus* NCFM binds to host mucin [55].

The probiotic strain *L. plantarum* KLDS1.0391 isolated from fermented dairy products has 293 genes encoding cell wall proteins. Fifty-two of the proteins are predicted to be potentially surface exposed, some of which have a confirmed role in adhesion, e.g. the moonlighting triose phosphate isomerase, elongation factor Tu, glyceraldehyde-3-phosphate dehydrogenase, three mucus binding proteins and three fibronectin-binding proteins [72]. Further, GADPH and triose phosphate isomerase were reported in *L. plantarum* 423 to play a role in adhesion and competitive exclusion of pathogens [73]. The effects of several moonlighting proteins, molecular chaperone proteins and other adhesion-related proteins have been confirmed, for example for S-layer proteins in *L. helveticus* strain T159 [50].

## 5. Effect of Compounds in the Diet on Adhesion of Lactobacilli

### 5.1. Prebiotics

The term “prebiotics” was introduced in the 1990s and defined as a “non-digestible food ingredient that beneficially affects the host by selectively stimulating the growth and/or activity of one or a limited number of bacteria already resident in the colon” [74]. This definition was first updated to state “selectively fermented ingredients that result in specific changes, in the composition and/or activity of the gastrointestinal microbiota, thus conferring benefit(s) upon host health” [75]. Recently, however, the prebiotic concept has been more broadly defined as “a substrate that is selectively utilized by host microorganisms conferring a health benefit” thus including non-carbohydrates as well as applications to non-GIT body sites and hence non-food substances [76]. Due to the rather low amounts in the Western-type diet, prebiotics are regarded as a sub-group of functional ingredients that can be added to foods such as yogurt, bread, cereals, biscuits, ice cream, etc. [75]. Prebiotic and other oligosaccharides in the food are metabolized by probiotic [77] and some commensal bacteria for example the prominent Bacteriodetes [78]. However, to be approved as a prebiotic destined as in the present case for the GIT, a candidate is expected also to fulfill the following criteria [79], i) be resistant to gastric pH, non-digestible by enzymes of the mammalian host, and not adsorbed in the GIT, ii) be fermentable by the GIT microbiota and prominently by probiotic bacteria, and iii) selectively stimulate growth and/or activity of intestinal bacteria correlated with health and well-being. Notably, certain commensal bacteria show specific beneficial behavior, including adhesion to the GIT epithelium, and some may be considered as prospective probiotics [24]. Similarly, results of in vitro studies have disclosed carbohydrate sources and other beneficial compounds which may be categorized as candidate prebiotics, but for which the validation by effect in human intervention trials has not been undertaken. Claims of status as prebiotics always require that health benefits have been achieved in controlled studies in the target host [76].

### 5.2. Synbiotics

Recently, attention has as well been on synbiotics, which are defined as “a mixture of probiotics and prebiotics that beneficially affects the host by improving the survival and implantation of live microbial dietary supplements in the gastrointestinal tract, by selectively stimulating the growth and/or activating the metabolism of one or a limited number of health promoting bacteria, and thus improving host welfare” [4,27,74]. The effect of the prebiotic in question should be specific to the probiotic, rendering a competitive advantage over other microbiota members, and the combination should have beneficial effects on the host. With regard to this concept, a few studies have been conducted to confirm effects of specific synbiotics under different perspectives, such as inflammatory bowel disease (IBD), irritable bowel syndrome, or colon cancer [4,80,81].

There are a number of studies, where the impact on the adhesion of probiotics grown on selected carbohydrate sources or other dietary components, has been investigated in vitro and which not necessarily comply with the rigorous definition of synbiotics as a mixture of prebiotics with approved effects in the host and probiotics. These cases are not involving approved synbiotics, but combinations of probiotics and dietary compounds which are of synbiotic-like character. Thus using fructose as a carbon source improved the adhesion to porcine gastric mucin for strains of *Lactobacillus delbrueckii* subsp. *bulgaricus* isolated from traditional Mongolian fermented milk products as compared to when grown on lactose, galactose or xylose [82]. Interest has also been on the mechanism of beneficial effects of plant polyphenols on probiotics for gut microbiota and human health, some of which (e.g., tannic acids) were proposed as prebiotics [83,84]. Finally, recently the presence of different polyunsaturated fatty acids was shown to reduce survival of lactobacilli, but still to increase their adhesion to intestinal cells [49].

## 6. Dietary Nutrients-Lactobacillus Interactions Characterized by Adherence and Proteome Analyses

We focused on adhesion and proteome changes to expand the molecular level insight into the mechanism of approved synbiotics and of candidate synbiotics. It is hypothesized that the utilization of oligosaccharides can modify the abundance of probiotic bacterial proteins important for adhesion to the GIT mucosa. Adhesion to mucin or HT-29 cells was assayed essentially according to the method based on labelling of bacteria with a fluorophore, allowing quantification of adhered cells [51] as reported for *L. acidophilus* NCFM [44,45,85] (refer to Section 4.1).

*Lactobacillus acidophilus* NCFM grown on different carbon sources, including approved and emerging prebiotics, and using glucose as reference, demonstrated a connection between the in vitro adhesion capacity to a mucin coating or a confluent HT-29 cell layer, respectively, (Table 1) and relative abundancy changes in bacterial proteomes (Table 2) [44,45,85]. The carbon sources included raffinose [44], fructooligosaccharides (FOS), galactooligosaccharides (GOS), lactulose, cellobiose, melibiose, palatinose, trehalose, and polydextrose [45]. Moreover, glucose media were supplemented with either mucin [45,86] or one of four plant polyphenols, resveratrol, tannic acid, caffeic acid, and ferulic acid [85]. In the set-up applied to assess adhesion, 2−3% of the bacterial cells from the reference culture on glucose adhered, while growth on various carbon sources and supplementation with mucin or plant polyphenols stimulated adhesion to reach 11% of the added cells. Notably, the levels of growth and adhesion to mucin or HT-29 cells layers were not directly coupled [44,45]. Thus *L. acidophilus* NCFM growth was stimulated on some and slowed by other media compared to glucose, moreover several carbon sources, GOS, lactulose, melibiose, palatinose, trehalose of which GOS and lactulose are approved as prebiotics [76], did not lead to improved adhesion [45]. The bacteria grew slowly on raffinose and cellobiose, but still exhibited elevated adherence to both mucin and HT-29 cells. Similar effects were found with polydextrose and FOS as carbon sources. Growth was stimulated by supplementation with mucin and also accompanied by improved adhesion, albeit only to HT-29 cells [45]. The effect on adhesion of selected plant polyphenols depended on their concentration in the growth media [45] (Table 1). Another study reported that wine polyphenols increased adhesion of probiotics to Caco-2 cells [86].

The relative protein abundancy changes were determined by comparative 2-DE for whole cell or surface proteomes of *L. acidophilus* NCFM cultured with the different carbon sources and supplements as compared to glucose followed by protein identification by in-gel digestion and mass spectrometry for selected spots undergoing relative intensity changes [44,45,85]. The findings by this straightforward procedure indicated selected stimulation of the occurrence of potential protein factors promoting adhesion. Some proteins of the surface proteome increased in relative abundancy by up to 4.4 fold, while others decreased by as much as 2.7 fold (Table 2). Notably, depending on the carbon source, a number of moonlighting proteins, elongation factor G, pyruvate kinase and fructose bis-phosphate aldolase increased in relative abundancy, whereas other moonlighting proteins decreased, including the well-known glyceraldehyde-3-phosphate dehydrogenase (GAPDH), triose phosphate isomerase, elongation factor Tu, phosphoglycerate kinase, molecular chaperone GroEL, and 6-phosphofructo kinase. Elongation factor G increased 2.1 and 2.4 fold with raffinose [44] and cellobiose [45], respectively, as carbon source, and decreased 3.5 and 2.0 fold when grown on glucose in the presence of either mucin or tannic acid (Table 2) [45,85]. Pyruvate kinase increased in the presence of mucin or resveratrol, but decreased when grown on glucose in the presence of ferulic acid or with cellobiose as carbon source [85] (Table 2). Thus effects on known moonlighting proteins can vary, and although it a priori may be expected that increased adhesion would be accompanied by increase in amounts of the affected moonlighting proteins, this was observed not always to be the case (Table 2). Indeed, the complexity is larger than analyzed for here as is also reflected by the fact that some proteins of altered abundancy occur in different proteoforms, for which knowledge is lacking on the eventual posttranslational modifications. For example, proteoforms originating from the same GADPH gene are seen by 2-DE surface proteome analysis of a small spot-train with a range of pI values and constant molecular mass [44,45,85].

Growth of *L. acidophilus* NCFM on lactulose (an approved prebiotic), raffinose or cellobiose elicited abundancy changes in the whole cell proteome indicating changes in the uptake and metabolism of these carbohydrates [44,87,88]. On cellobiose, this included several moonlighting proteins, phosphoglycerate kinase, triose phosphate isomerase, and GADPH as well as surface layer protein [88]. A human intervention study demonstrated the putative synbiotic of *L. acidophilus* NCFM and cellobiose to increase the abundance of lactobacilli, bifidobacteria, and branched chain fatty acids without affecting the human gut bacterial diversity and the amount of short chain fatty acids [89]. By contrast, however, in in vitro colon simulator experiments, production of the beneficial short chain fatty acids and the count of *L. acidophilus* NCFM increased for synbiotics candidates containing cellobiose or raffinose [90]. Investigation of the emerging prebiotic raffinose, for which a rich source is side streams in soy protein production, was motivated by the lack of α-galactosidase in the human GIT and demonstrated to increase adhesion of *L. acidophilus* NCFM on both mucin and HT-29 cells by 3 fold (Table 1; Figure 4) [44].

## 7. Conclusions and Future Perspectives

Understanding of the possible mechanisms by which food components influence on adhesion of probiotic bacteria in vivo can benefit from thorough investigation at the molecular level in vitro. The present mini-review provides insights into the relationship between surface-proteins, carbon source and adhesion ability of probiotic bacteria with emphasis on *L. acidophilus* NCFM. The findings demonstrate a connection between dietary nutrients, probiotic adhesion and protein profiles. Although this set-up is an oversimplification of the in vivo situation, which likely has distinct uncovered characteristics, the in vitro experiments illustrate both the complexity and that knowledge is lacking on molecular details of GIT adhesion and its potential implications for the host. The described reactions of *L. acidophilus* NCFM to growth conditions (carbon source and supplementation with mucin or plant polyphenols) contribute to decoding the intricate interplay of probiotics, diet and host in particular with relevance for synbiotics. The recognition of beneficial health effects elicited by combinations of microorganisms and dietary components continues to include novel systems which deserve to be explored at the molecular level by using proteomics strategies. It is for example realized recently that different bacterial β-glucans stimulate adhesion [91,92]. Additionally, molecular level characterization will be relevant for the effect of well-known beneficial components such as cereal β-glucans as well as other dietary fibers and their oligosaccharide degradation products. Still however, only clinical studies will be able to provide the basis for accepting the new dietary nutrients as prebiotic/synbiotics.

## Figures and Tables

**Table 1:** Change in adhesive capacity onto mucin coating and HT-29 cells, respectively, of *Lactobacillus acidophilus* NCFM grown on different carbon sources and supplements as compared to growth on glucose.

Carbon Source (1%)/Supplementation	Mucin ^d^ (Fold-Increase)	HT-29 ^d^ (Fold-Increase)
Raffinose ^a^	3 (*p* < 0.0001)	3 (*p* < 0.0001)
Cellobiose ^b^	2.5 (*p* < 0.05)	2.5 (*p* < 0.05)
Polydextrose ^b^	3 (*p* < 0.05)	2 (*p* < 0.05)
FOS ^b^	n.d.	2 (*p* < 0.05)
Glucose/mucin (0.1%) ^b^	n.d.	2 (*p* < 0.05)
Glucose/resveratrol ^c^	2.3 (*p* < 0.01) (100 µg/mL)	1.4 (*p* < 0.01) (100 µg/mL) 3.0 (*p* < 0.001) (250 µg/mL)
Glucose/tannic acid ^c^	0.4 (*p* < 0.001) (250 µg/mL)	5.0 (*p* < 0.001) (100 µg/mL)
Glucose/caffeic acid ^c^	1.3 (*p* < 0.05) (250 µg/mL)	n.d.
Glucose/ferulic acid ^c^	0.8 (*p* < 0.01) (100 µg/mL) 1.9 (*p* < 0.001) (250 µg/mL) 1.2 (*p* < 0.001) (500 µg/mL)	1.4 (*p* < 0.001) (250 µg/mL)

References: ^a^ [44], ^b^ [45], ^c^ [85]. ^d^ Statistic significance level of increased adhesion compared to growth on glucose as control and concentration of polyphenol during growth are both given in parentheses. n.d. = not detected.

**Table 2:** Surface proteins showing relative abundancy changes in *Lactobacillus acidophilus* NCFM with increased adhesive capacity.

Protein Name	Accession Number	Carbon Source and Protein Fold-Change						
		Ra ^a^	Ce ^b^	Mu ^b^	Re ^c^	TA ^c^	CA ^c^	FA ^c^
Phosphate starvation inducible protein stress-related	YP_193579	+4.4	+4.4					
Thermostable pullulanase	YP_194553	+2.3	+3.5					
Elongation factor G ^M^	YP_193213	+2.1	+2.4	−3.5		−2.0		
50S Ribosomal protein L7/L12	YP_193293				+2.0			
Pyruvate kinase ^M^	YP_193840		−1.7	+1.9	+2.1			−1.6
Fructose-bis-phosphate aldolase ^M^	YP_194445			+1.8				
Elongation factor P	YP_194511				+1.7		−1.7	
50S Ribosomal protein L22	YP_193220				+1.6			
Glutamyl tRNA synthase	YP_193270						+1.5	
Hypothetical protein LBA1769	YP_194608				+1.4			
Aminopeptidase	YP_104682					+1.4		
S-layer Protein	YP_193101	+1.3						
Glycoprotein endopeptidase	YP_193310	−1.4				+1.3		
6-phophofructokinase ^M^	YP_193839				−1.4			
Phosphoglycerate kinase ^M^	YP_193605	−1.4		−3.1				
Molecular chaperone GroEL ^M^	YP_193328	−1.5						
D-lactate dehydrogenase	YP_192990						−1.5	
50S Ribosomal protein L10	YP_193292	−1.6						
Trigger factor ^M^	YP_193738							−1.7
50S Ribosomal protein L1	YP_193283	−1.8						
ATP synthase FOF1 subunit alpha	YP_193673	−1.8						
Oligoribonuclease	YP_193337							−1.8
Elongation factor Tu ^M^	YP_193737	−1.8					−1.8	
Lysine tRNA ligase	YP_193205						−1.9	
L-lactate dehydrogenase	YP_193195							−1.6
Elongation factor Ts	YP_194131		−2.0					
Triose phosphate isomerase ^M^	YP_193606	−2.0					−2.0	−1.7
Manganese-dependent inorganic pyrophosphatase	YP_194000	−1.5		−2.1		−1.5		−1.5
Aspartate tRNA ligase	YP_193821						−2.2	
Adenylosuccinate synthase	YP_194721			−2.3	−1.5		−1.5	
30S Ribosomal protein S1 ^M^	YP_193850			−2.3			−1.8	−1.7
Ribonucleoside triphosphate reductase	YP_192977			−2.3				
Glyceraldehyde-3-p dehydrogenase ^M^ (GAPDH)	YP_193579	−2.4 −1.9 −1.6	−2.0 −1.5		−2.0 −1.6			
BipAEFTU family GTP-binding protein ^M^	YP_193724			−2.7				

^M^ Reported moonlighting proteins. References: ^a^ [44], ^b^ [45], ^c^ [85]. Carbon source, Ra = raffinose; Ce = cellobiose; Mu = glucose supplemented with mucin; Re = glucose in presence of resveratrol; TA = glucose in presence of tannic acid; CA = glucose in the presence of caffeic acid; FA = glucose in presence of ferulic acid.
